# Contributions of narrow- and broad-spiking prefrontal and parietal neurons on working memory tasks

**DOI:** 10.3389/fnsys.2024.1365622

**Published:** 2024-03-21

**Authors:** Rana Mozumder, Sophia Chung, Sihai Li, Christos Constantinidis

**Affiliations:** ^1^Department of Biomedical Engineering, Vanderbilt University, Nashville, TN, United States; ^2^Neuroscience Program, Vanderbilt University, Nashville, TN, United States; ^3^Department of Neurobiology, The University of Chicago, Chicago, IL, United States; ^4^Department of Ophthalmology and Visual Sciences, Vanderbilt University Medical Center, Nashville, TN, United States

**Keywords:** prefrontal cortex, posterior parietal cortex, monkey, neurophysiology, working memory

## Abstract

Neurons that generate persistent activity in the primate dorsolateral prefrontal and posterior parietal cortex have been shown to be predictive of behavior in working memory tasks, though subtle differences between them have been observed in how information is represented. The role of different neuron types in each of these areas has not been investigated at depth. We thus compared the activity of neurons classified as narrow-spiking, putative interneurons, and broad-spiking, putative pyramidal neurons, recorded from the dorsolateral prefrontal and posterior parietal cortex of male monkeys, to analyze their role in the maintenance of working memory. Our results demonstrate that narrow-spiking neurons are active during a range of tasks and generate persistent activity during the delay period over which stimuli need to be maintained in memory. Furthermore, the activity of narrow-spiking neurons was predictive of the subject’s recall no less than that of broad-spiking neurons, which are exclusively projection neurons in the cortex. Our results show that putative interneurons play an active role during the maintenance of working memory and shed light onto the fundamental neural circuits that determine subjects’ memories and judgments.

## Introduction

Working memory is the limited-capacity system of maintaining and manipulating information in current thought (Jaffe and Constantinidis, 2021). It is applicable in spatial, episodic, and verbal tasks (Baddeley, 2012) and can be improved with training (Qi and Constantinidis, 2013; Constantinidis and Klingberg, 2016). Lesion, neuroimaging, and neurophysiological studies have shown the dorsolateral prefrontal cortex (dlPFC) and the posterior parietal cortex (PPC) to be two key brain regions that give rise to this cognitive ability (Constantinidis and Klingberg, 2016). Analysis of neurophysiological recordings during visuospatial working memory tasks, such as the oculomotor delayed-response task, revealed that neurons generate persistent activity which represents information regarding the location of visual stimuli, thus providing a neural correlate of working memory (Riley and Constantinidis, 2016). The bump attractor model provides mechanistic insights on how persistent activity can be maintained, by virtue of recurrent excitation between neurons, and accounts for behavior in working memory task, tying fluctuations of persistent firing rate to variability of responses (Compte et al., 2000; Wimmer et al., 2014). However, the neural mechanisms from which working memory arises are a continuing debate in the field. Competing theories have questioned whether persistent activity is present across all tasks, suggesting instead, that “activity-silent” models can better account for working memory (Stokes, 2015). Other models have posited that the rhythmic spiking of neurons is the critical neural variable, instead, so that each attractor state is accompanied by bursts of gamma oscillations, resulting from fast, local feedback inhibition (Lundqvist et al., 2016).

To adjudicate between competing models, it is thus imperative to determine whether proposed neural correlates of working memory can account for behavior across different cognitive tasks, and similarly, whether model predictions hold across tasks. In a recent study Li et al. tested whether persistent activity could predict subtle changes in what a subject recalls and how this activity differed in dlPFC and PPC (Li et al., 2021). This study used a novel working memory task that dissociated motor preparation from spatial working memory. Neurons in the dlPFC and PPC neurons were shown to be equally predictive of behavior but there were still subtle differences between the two areas. In a second study of interest, Qi et al. trained monkeys to remember either the first or second of two stimuli presented in sequence (Qi et al., 2015). This study, too, revealed that neurons with persistent activity, particularly in the dlPFC can account for what information the subjects represented in memory.

These studies examined activity pooled from all neurons in these areas, however it is known that prefrontal pyramidal neurons and interneurons can play distinct roles during cognitive tasks (Hussar and Pasternak, 2009; Ardid et al., 2015; Jacob et al., 2016). The bump attractor model predicts that persistent activity is maintained by virtue of structured connections between pyramidal neurons and interneurons and that both populations generate tuned persistent activity that should be predictive of behavior (Compte et al., 2000). A specialization of interneuron function inconsistent with this prediction would cast doubt on the validity of the bump attractor model as the primary mechanism of working memory. Different neuron types can be classified from extracellular recordings based on the waveform of their action potentials as narrow-spiking (NS) with shorter action potentials and broad-spiking (BS) with longer action potentials, sometimes referred to as fast-spiking and regular-spiking respectively, as well (Constantinidis and Goldman-Rakic, 2002; Trainito et al., 2019). We were therefore motivated to determine whether NS neurons generate persistent activity across tasks and brain areas, and whether their such persistent activity is predictive of behavior, consistent with the bump attractor model.

## Materials and methods

The following methods are summarized from the Li et al. (2021) and Qi et al. (2015) studies. Four male rhesus monkeys (*Macaca mulatta*) were used in these experiments. Neural recordings were carried out in areas 8 and 46 of the dorsolateral prefrontal cortex and areas 7a and lateral intraparietal area (LIP) of the posterior parietal cortex. All experimental procedures followed guidelines by the U.S. Public Health Service Policy on Humane Care and Use of Laboratory Animals and the National Research Council’s Guide for the Care and Use of Laboratory Animals were reviewed and approved by the Wake Forest University Institutional Animal Care and Use Committee.

### Experiment setup

Monkeys sat in a primate chair with their head fixed while viewing a liquid crystal display monitor. Animals fixated on a white square in the center of the monitor screen. Animals were required to fixate on a 0.2° spot appearing in the center of the monitor screen and maintain fixation within a 3° window. During each trial, the animals maintained fixation on the spot while visual stimuli were presented at peripheral locations. Any break of fixation terminated the trial, and no reward was given. Eye position was monitored throughout the trial using a non-invasive, infrared eye position scanning system (model RK-716; ISCAN, Burlington, MA). Eye position was sampled at 240 Hz, digitized, and recorded. Visual stimuli display, monitoring of eye position, and the synchronization of stimuli with neurophysiological data were performed with in-house software (Meyer and Constantinidis, 2005) implemented in MATLAB (Mathworks, Natick, MA).

### Behavioral tasks

**Figure 1:**
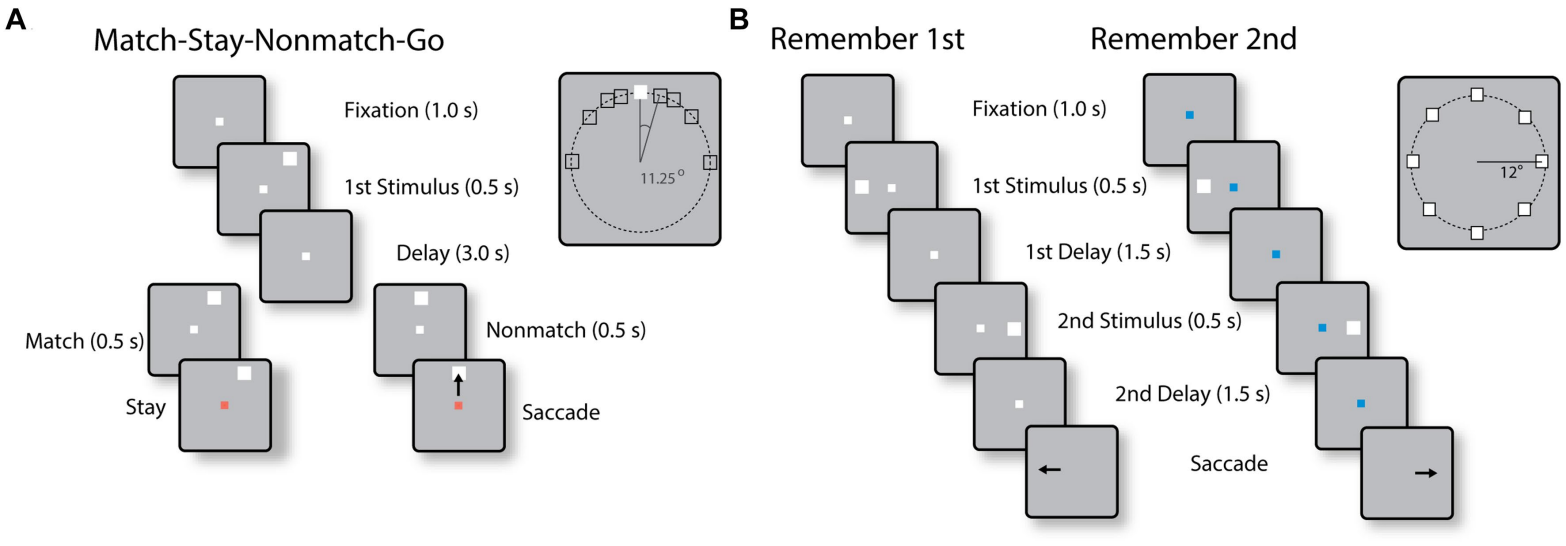
**(A)** Sequence of events in the MSNG task. The monkey is required to observe the cue and maintain fixation during the delay period. If a second stimulus appears at the same location as the cue (match), the monkey needs to stay at the fixation point after its color changes; if it deviates (nonmatch), the monkey is required to make an eye movement to the second stimulus once the color of the fixation point changes. **(B)** Sequence of events in the R1R2 task. In the remember-first task, the fixation point is white, and the monkey is required to make an eye movement to the first stimulus, regardless of the location of a second stimulus, which is a distractor. In the remember-second task, the first stimulus is now a distractor and the monkey is required to make an eye movement to the remembered location of the second stimulus.

Two monkeys were trained to perform the Match-Stay Nonmatch-Go (MSNG) task (Figure 1A). The task required the monkeys to remember the location of a cue. After a 3 s delay period, a second stimulus appeared, either at the identical location (match) or a different location (nonmatch). After 500 ms, the fixation point changed color. If the second stimulus was a match, the monkey was required to maintain fixation; if the second stimulus was a nonmatch, the monkey was required to make a saccade towards this visible stimulus. The monkeys received a liquid reward for each correct response. Possible cue locations included a reference location (white square in the inset of Figure 1A) and eight locations deviating from the reference location by an angular distance of 11.25°, 22.5°, 45° and 90°, clockwise and counterclockwise. In each daily session, the cue could appear pseudo-randomly at one of the eight possible locations. Since only a limited range of stimulus locations was explored in each session, an effort was made to position stimuli based on the estimated best neuronal responding location of neurons isolated in real-time, however we recorded neuronal activity with multiple electrode arrays, and the location of the stimuli could fall at any position relative to a neuron’s receptive field. The use of this range of conditions allowed us to randomly interleave trials that differed considerably in difficulty. Error trials were eventually used to determine the relationship between neural activity and behavior. The cue was followed by a matching stimulus appearing at the same location as the cue in approximately half the trials or by a nonmatch stimulus, which could only appear at the reference location. The reference location varied from session to session.

Two different monkeys were trained to perform the Remember First – Remember Second (R1R2) task (Figure 1B). In this task, two stimuli also appeared in sequence, with intervening delay periods between them, now requiring the monkey to remember and make an eye movement to either the first or the second stimulus according to the color of the fixation point. The monkeys were required to saccade to the location of the first stimulus if the fixation point was white in color (remember-first condition), and to the location of the second stimulus if the fixation point was blue (remember-second condition). To minimize confusion about the stimulus to be remembered, trials with white and blue fixation points were presented in blocks.

### Surgery and neurophysiology

Two, 20-mm diameter craniotomies were performed over the lateral prefrontal cortex and the posterior parietal cortex, and a recording cylinder was implanted over each site. Neurophysiological recordings were obtained as described before (Zhou et al., 2016). Tungsten-coated electrodes with a 200 or 250 μm diameter and 4 MΩ impedance at 1 kHz were used (FHC, Bowdoinham, ME). Arrays of up to 4-microelectrodes spaced 0.5–1 mm apart were advanced into the cortex with a Microdrive system (EPS drive, Alpha-Omega Engineering) through the dura into the cortex.

### Neural data analysis

Data analysis was performed in the MATLAB computational environment (Mathworks, Natick, MA, version 2019-2022a). Recorded spike waveforms were sorted into separate units using a semi-automated cluster analysis process of the KlustaKwik algorithm (Harris et al., 2000). Action potential waveforms of all neurons were fitted with a smooth function, using a Generalized Additive Model, to avoid aliasing. This analysis was performed in R (version 4.3.1), using the GAM function of the mgcv package. We used cubic regression to fit the original waveform points and subsequently up-sampled, using the spline function. The time difference between the trough and subsequent peak of the waveform was then identified as the spike width, similar to previous studies (Womelsdorf et al., 2014; Trainito et al., 2019). Neurons were classified as narrow-spiking (NS) putative interneurons or broad-spiking (BS) putative pyramidal neurons based on this spike width.

Neurons generating persistent activity were identified as those with firing rates during the (first) delay period that were higher compared to the 1 s baseline fixation period that preceded the cue presentation, based on a paired *t*-test, evaluated at the *p* < 0.05 level. This analysis was performed based on correct trials, only. Population discharge rates were evaluated by averaging activity from multiple neurons and constructing Peri-Stimulus Time Histograms (PSTH). These were constructed using the best stimulus (preferred cue) for each neuron. Cohen’s d was used to estimate effect sizes. Correct and error conditions were compared for trials also involving the preferred cue of each neuron. Neurons with at least two error trials in this condition were included in analysis. Variability of neuronal responses was quantified by computing the Fano factor (variance divided by the mean) of spike counts in the delay period (Qi and Constantinidis, 2012).

To quantify the trial-to-trial association between perceptual choice and neuronal activity, we analyzed trials involving the best and most distant stimulus location, and trials that resulted in correct choices and incorrect choices, using Receiver Operating Characteristic (ROC) analysis (Britten et al., 1996; Mendoza-Halliday et al., 2014). Firing rates of trials involving the same sequences of stimuli were pooled separately for correct and error outcomes. An ROC curve was computed from these two distributions of firing rates. The area under the ROC curve is referred to in the perceptual inference literature as “choice probability” and represents a measure of correlation between the behavioral choice and neuronal activity. A value of 1 indicates a perfect correlation between the behavioral choices and the neuronal discharge rates; a value of 0.5 indicates no correlation between the two. Time-resolved choice probabilities were computed from the spikes in 500 ms time windows, stepped by 50 ms intervals. Results from all available neurons were averaged together to produce population responses.

## Results

### Properties of NS and BS neurons in the dlPFC and PPC

**Figure 2:**
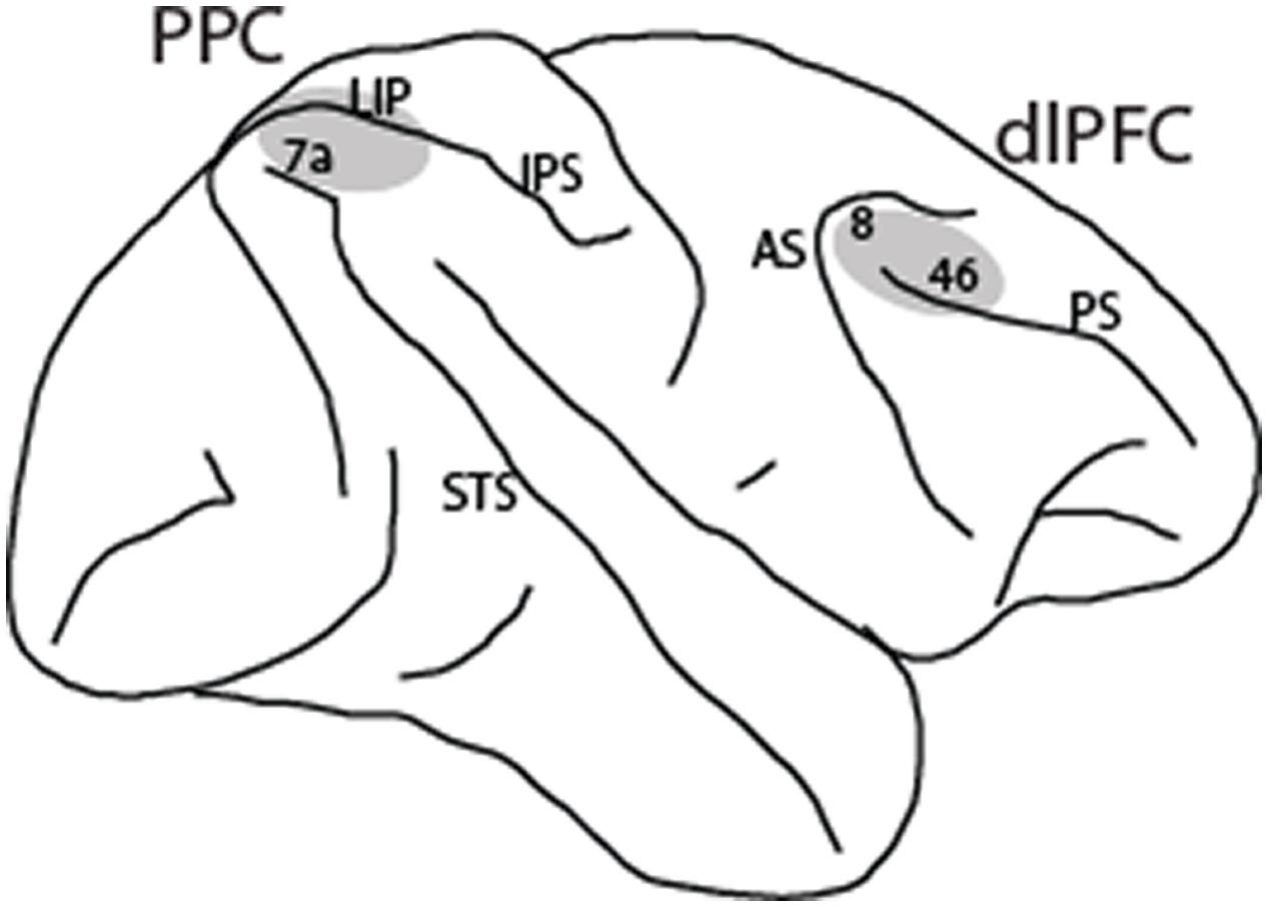
Regions of neurophysiological recordings, comprising areas 8 and 46 in the dorsolateral prefrontal cortex (dlPFC) and areas 7a and LIP in the posterior parietal cortex (PPC). IPS, intraparietal sulcus; STS, superior temporal sulcus; AS, arcuate sulcus; PS, principal sulcus.

**Figure 3:**
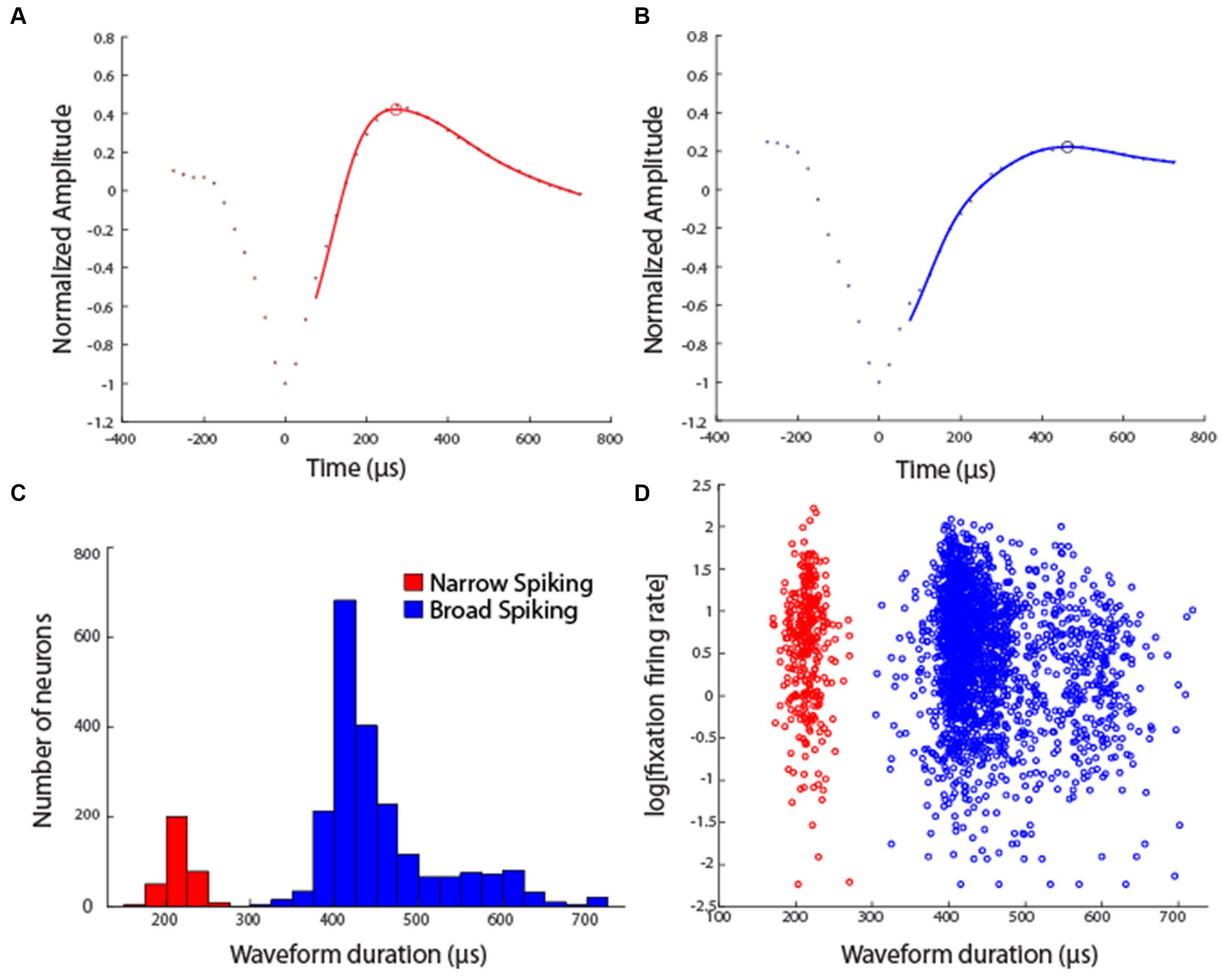
**(A)** Example waveform of a single Narrow Spiking unit. Points represent average normalized voltage value across all waveforms of this unit. Line represents fitted generalized additive model through experimental points. Open circle represents the estimated peak of the waveform. **(B)** Example waveform of a single Broad Spiking unit. **(C)** Distribution of spike widths among all neurons in the sample, across areas and tasks (sample size of 341 NS units and 2,120 BS units). **(D)** Firing rate, computed in the fixation interval of the task is plotted against the waveform duration of each unit.

Neuronal activity was recorded from areas 8 and 46 of the dorsolateral prefrontal cortex and areas 7a and LIP of the posterior parietal cortex (Figure 2) in four monkeys. Two of the monkeys were trained to perform the MSNG task (Figure 1A). A total of 577 neurons were recorded from the dlPFC and 859 neurons were recorded from the PPC in this task. Two additional monkeys were trained to perform the R1R2 task (Figure 1B). A total of 423 neurons were recorded from the dlPFC and 602 neurons were recorded from the PPC in this task. Distribution of spike widths of these neurons exhibited clear bimodal distribution (Hartigan’s dip test: dip value = 0.042, *p* < 0.001). Neurons with spike widths ≤300 μs were thus classified as narrow-spiking (NS) putative interneurons, and neurons with spike widths >300 μs were classified as broad-spiking (BS) putative pyramidal neurons (Figure 3). From the data recorded with the MSNG task, 47 dlPFC neurons (8%) and 136 PPC neurons were classified as NS (15.8%). For the data recorded with the R1R2 task, 66 dlPFC neurons (15.6%) and 92 PPC neurons were classified as NS (15.3%). Mean firing rate, computed in the baseline, fixation period of the tasks was overall higher in the NS population (10.28 spikes/s) than the BS population (8.93 spikes/s). Although considerable variability was present in both populations, the difference did reach statistical significance (Wilcoxon rank-sum test, *p* = 0.01). The overall percentage of NS neurons in our sample (341/2461, 14%) was lower than the percentage of interneurons identified by anatomical studies, generally estimated in the 20–30% range, however this includes non-fast spiking interneuron cell types (Markram et al., 2004; Torres-Gomez et al., 2020). Prior neurophysiological studies in the monkey prefrontal cortex from other laboratories have classified approximately 13–20% of neurons as narrow spiking (Diester and Nieder, 2008; Johnston et al., 2009; Ardid et al., 2015), generally consistent with the percentage we report here.

We focused particularly on neurons that exhibited significantly elevated responses in the first cue period or the delay period in the MSNG task, compared with the baseline activity (paired *t*-test, *p* < 0.05) as these were identified in Li et al. (2021). A total of 144 neurons with such activity were identified in dlPFC (49 in subject KE, and 95 in subject LE) and 145 neurons in the PPC (44 in subject KE, and 101 in subject LE). Of those, 12 dlPFC neurons were identified as NS (8.3%); similarly, 28 PPC neurons were identified as NS (19.3%). The proportion of neurons that was classified as NS did not differ significantly between the task-responsive and non-task responsive neurons for either the dlPFC (two-tailed Fisher’s exact test: *p* = 1.0) or the PPC (two-tailed Fisher’s exact test: *p* = 0.21).

We also examined task responsive neurons in the R1R2 task, as these were identified in the Qi et al. study, as neurons that exhibited significantly elevated responses in the first delay period compared with the baseline activity (paired *t*-test, *p* < 0.05). A total of 182 neurons with persistent activity were identified in the dlPFC (38 in subject GR, and 144 in subject HE) and 180 neurons in the PPC (86 in subject GR, and 94 in subject HE). Of those 28 dlPFC neurons were identified in NS (15.4%); similarly, 29 PPC neurons were identified as NS (16.1%). For this task too, the proportion of neurons that was classified as NS did not differ significantly between the task-responsive and non-task responsive neurons for either the dlPFC (two-tailed Fisher’s exact test: *p* = 1.0) or PPC (two-tailed Fisher’s exact test: *p* = 0.71).

**Figure 4:**
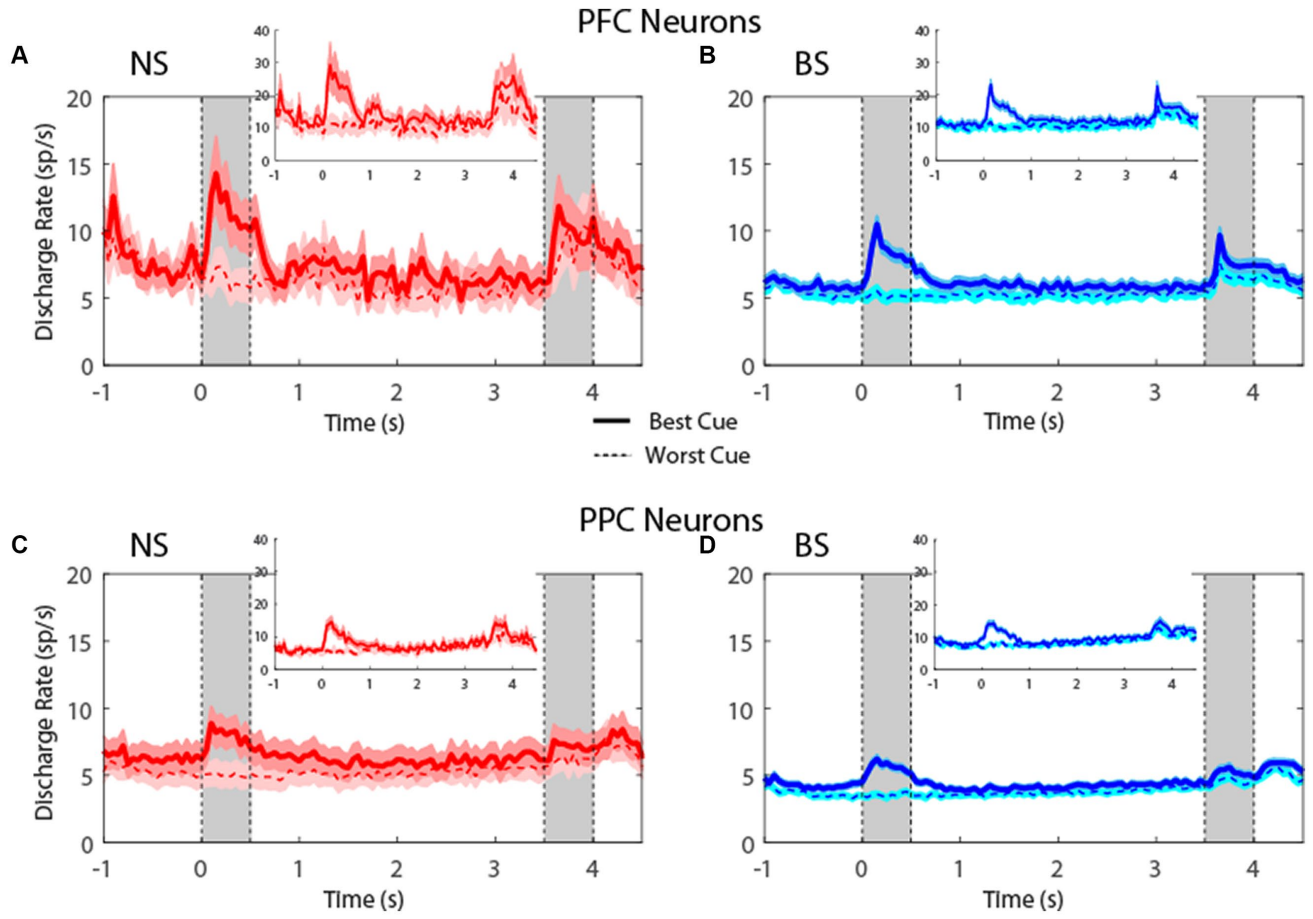
**(A)** Population PSTH showing mean firing rate from narrow-spiking dlPFC neurons recorded in the MSNG task (*n* = 47). Inset, mean firing rate of neurons with significantly elevated responses (*n* = 12). Gray bars indicate time of appearance of the two stimuli in the task (0–0.5 s and 3.5–4 s). Time of −1 s represents the beginning of the fixation period. The solid traces indicate average activity of neurons when the stimulus appeared in location that elicited the best cue response (best cue), while the dotted lines indicate neuronal activity when the stimulus appeared at the most distant location from the preferred location (worst cue). Shaded areas indicate standard error of the mean (SEM). **(B)** As in **A**, for broad-spiking dlPFC neurons (*n* = 530). Inset, mean firing rate of neurons with significantly elevated responses (*n* = 132). **(C)** Averaged PSTH of neuronal spike discharges from narrow-spiking PPC neurons recorded in the MSNG task (*n* = 136). Inset, mean firing rate of neurons with significantly elevated responses (*n* = 28). **(D)** As in **C**, for broad-spiking PPC neurons (*n* = 723). Inset, mean firing rate of neurons with significantly elevated responses (*n* = 117).

Plotting the firing rate of all neurons in the MSNG task (Figure 4) revealed that NS neurons generated robust responses to stimuli. In the MSNG Task, dlPFC mean firing rates did not reach a significant difference between the NS and BS neurons in either the cue (*t*-test: *t*_575_ = 1.56, *p* = 0.12, effect size = 0.23) or the delay period (*t*-test: *t*_575_ = 0.45, *p* = 0.66, effect size = 0.07). NS neurons in the PPC had significantly higher firing rate than BS neurons for both the cue (*t*-test: *t*_857_ = 2.7, *p* = 0.007, effect size = 0.19) and delay period (*t*-test: *t*_857_ = 2.61, *p* = 0.009, effect size = 0.17). Results were very similar when we considered the populations of neurons with significantly elevated activity during the task (insets of Figure 4).

**Figure 5:**
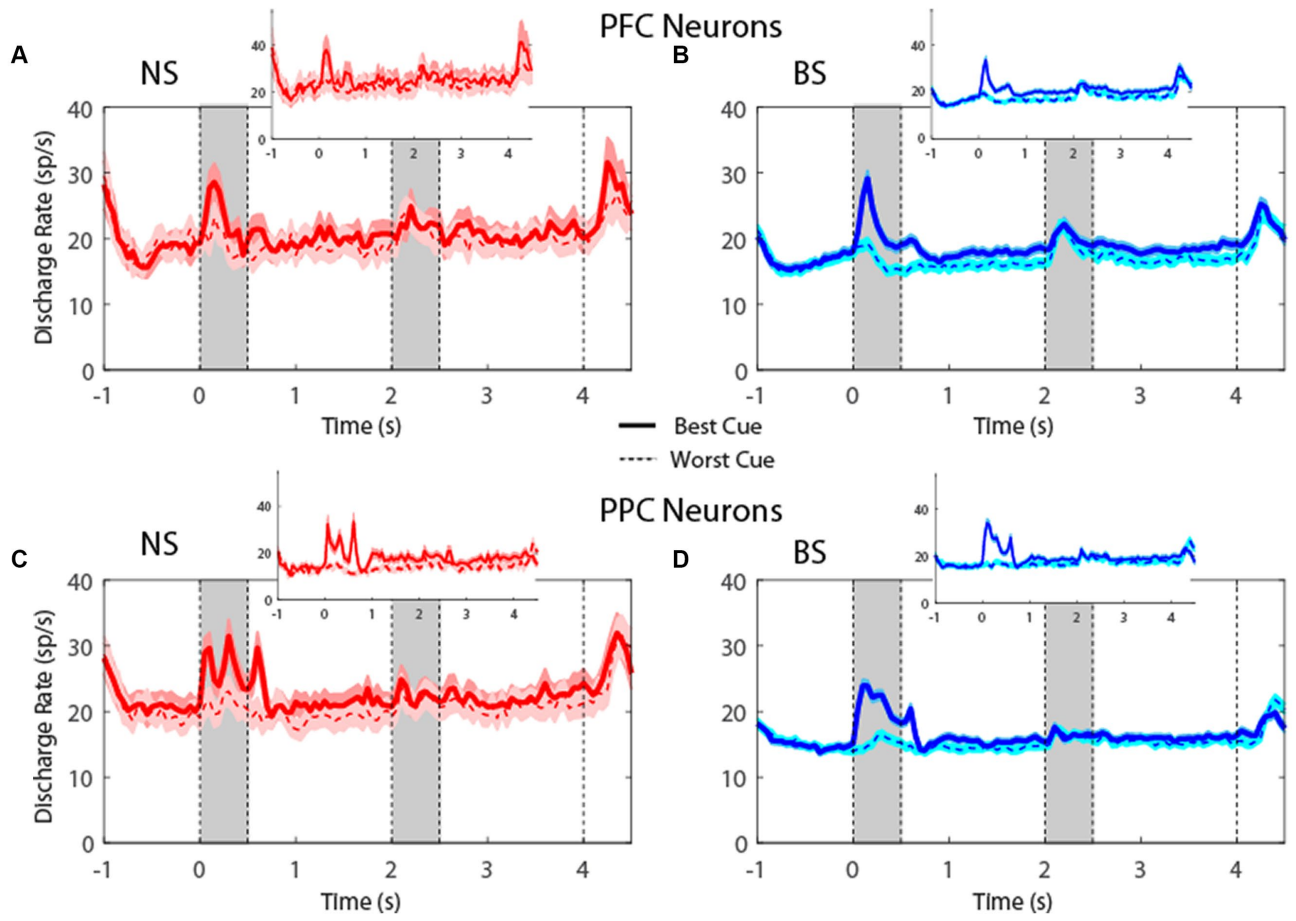
**(A)** Population PSTH showing mean firing rate from narrow-spiking dlPFC neurons recorded in the R1R2 task (*n* = 66). Inset, mean firing rate of neurons with significantly elevated responses (*n* = 28). Gray bars indicate time of appearance of the two stimuli; right-most dotted line indicates offset of fixation point that signals the beginning of the saccade period (0–0.5 s, 2–2.5 s, and 4–4.5 s). Time − 1 s represents the beginning of the fixation period. Solid lines indicate neuronal activity for the best location (based on mean cue firing rate), while the dotted lines indicate activity when the cue appeared at the location diametric to the best location. Shaded areas indicate standard error of mean (SEM). **(B)** Same as in **A**, for broad-spiking dlPFC neurons (*n* = 357). Inset, mean firing rate of neurons with significantly elevated responses (*n* = 154). **(C)** Averaged PSTH of neuronal spike discharges from narrow-spiking PPC neurons recorded in the R1R2 task (*n* = 92). Inset, mean firing rate of neurons with significantly elevated responses (*n* = 29). **(D)** Same as in **C**, for broad-spiking PPC neurons (*n* = 510). Inset, mean firing rate of neurons with significantly elevated responses (*n* = 151).

For the R1R2 task (Figure 5), the firing rate for NS neurons were not significantly different than BS neurons, neither in cue (*t*-test: *t*_421_ = 0.61, *p* = 0.54, effect size = 0.09 for dlPFC; *t*-test: *t*_600_ = 0.69, *p* = 0.49, effect size = 0.08 for PPC) nor in delay period (*t*-test: *t*_421_ = 0.19, *p* = 0.85, effect size = 0.03 for dlPFC; *t*-test: *t*_600_ = 0.48, *p* = 0.63, effect size = 0.05 for PPC). In this case, too, results were similar when we considered the populations of neurons with significantly elevated activity during the task (insets in Figure 5). The absolute firing rate was higher overall in the R1R2 task than the MSNG task, and this was true for both NS and BS neurons. This likely reflects differences between monkeys tested in the two studies and subtle experimental differences (e.g., more emphasis on selection of task-responsive neurons for the purposes of the R1R2 study).

Examining neuronal responses of Figures 4, 5 created the appearance that variability of firing rate was greater for NS than BS neurons, however, that was a consequence of the smaller NS sample size. When we calculated the Fano factor (variance divided by mean) of spike counts in the delay period, the result revealed similar Fano factor values for NS neurons in the MSNG task (PFC NS = 1.18, PFC BS = 1.11, PPC NS = 1.29, PPC BS = 1.17), and in the R1R2 task (PFC NS = 1.31, PFC BS = 1.38, PPC NS = 1.48, PPC BS = 1.47). Only the difference between NS and BS neurons in the PPC for the MSNG task reached statistical significance (Wilcoxon rank-sum test, *p* = 0.025). All other tests produced values *p* > 0.1. This analysis did confirm lower variability in the prefrontal than the posterior parietal cortex, as we have described previously (Qi and Constantinidis, 2015).

Overall, the time courses of activity were very similar between NS and BS neurons in the two tasks, and in both dlPFC and PPC. These results indicate that comparable percentages of NS and BS neurons exhibited task related activity in the context of these working memory tasks; firing rates of NS and BS populations generally mirrored each other in the context of these tasks, although NS neurons exhibited generally higher firing rate, particularly in the PPC.

### ROC analysis

**Figure 6:**
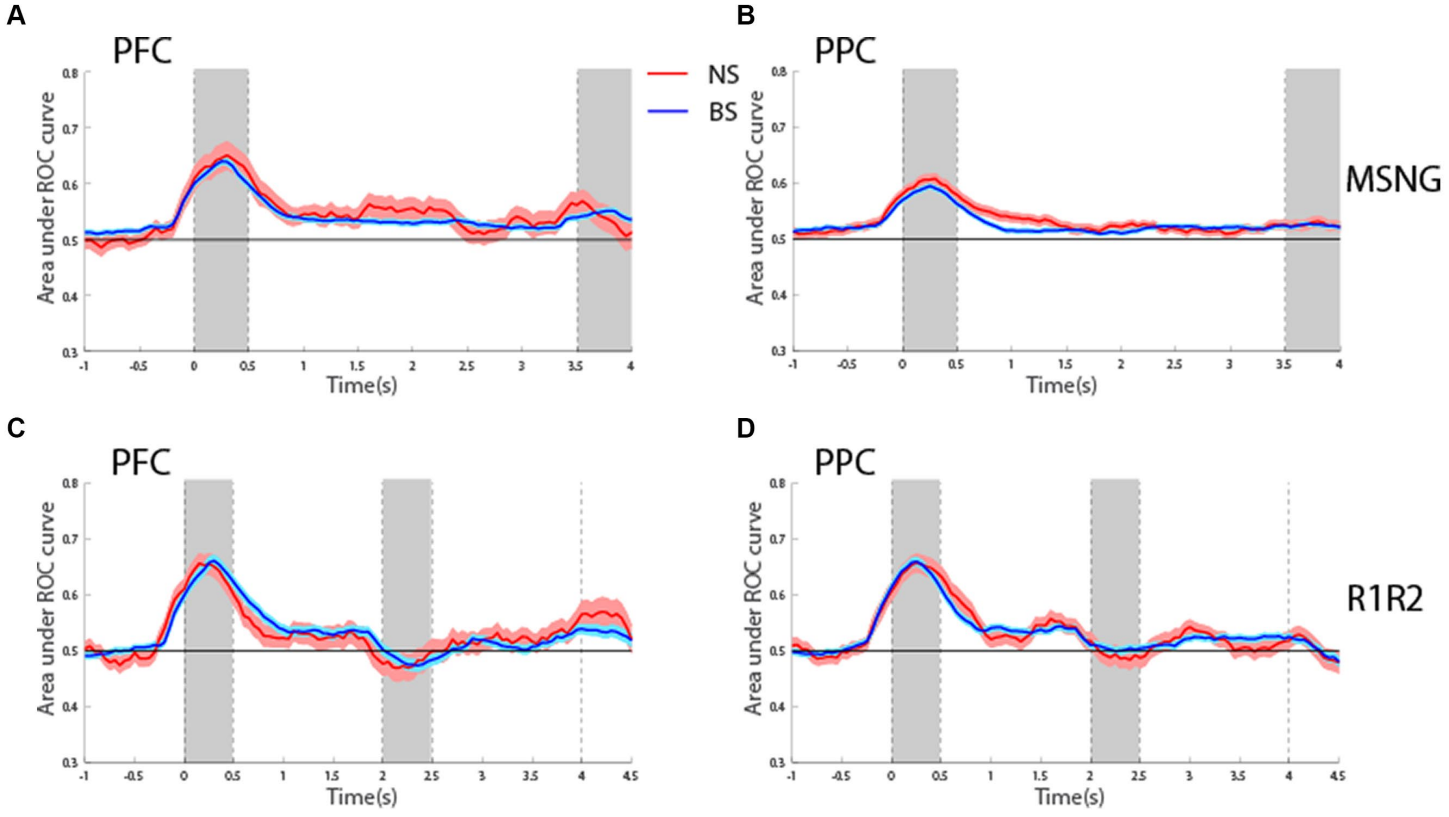
**(A)** Averaged area under the ROC curve from NS (red line, *n* = 41) and BS (blue line, *n* = 526) neurons recorded from the dlPFC plotted as a function of time for the MSNG task. Each curve represents difference in firing rate distributions between the best and most distant location. Shaded area represents SEM. **(B)** As in **A** for NS (red line, *n* = 136) and BS (blue line, *n* = 717) neurons recorded from the PPC in the MSNG task. **(C)** Averaged area under the ROC curve from NS (red line, *n* = 66) and BS (blue line, *n* = 352) neurons recorded from the dlPFC in the R1R2 task. **(D)** As in **C**, for NS (red line, *n* = 92) and BS (blue line, *n* = 504) neurons recorded from the PPC in the R1R2 task.

To assess the reliability with which firing rates of BS and NS units could predict the visual stimulus and the subject’s choice, we performed Receiver Operating Characteristic (ROC) analysis. We first considered the area under the ROC curve, comparing the distribution of firing rates for the best location of its neuron and its more distant one (diametric in the case of the R1R2 task). This analysis was performed in a time-resolved fashion and tested whether firing rates of both BS and NS units discriminated between the spatial locations of the stimuli during the delay period better than chance, represented by ROC values equal to 0.5 (Figure 6). For the MSNG task, BS units exhibited mean ROC values significantly higher than chance (one sample *t*-test, *t*_525_ = 10.1, *p* = 3.56E-22, effect size = 0.44 for dlPFC; *t*_716_ = 10.1, *p* = 1.24E-22, effect size = 0.38, for PPC). This result was in agreement with our previous studies (Li et al., 2021), and was expected, since the majority of neurons in the sample were BS neurons. Importantly, NS units also exhibited significantly elevated mean ROC values over the delay period (one sample *t*-test, *t*_40_ = 2.95, *p* = 0.005, effect size = 0.46; *t*_135_ = 5.09, *p* = 1.18E-6, effect size = 0.44, for dlPFC and PPC, respectively). In fact, the time course and mean ROC values of the two populations were virtually identical (Figures 6A,B) and no significant difference was present between the mean ROC values of the BS and NS populations during the delay period (*t*-test, *t*_565_ = 0.77, *p* = 0.44, effect size = 0.11 for dlPFC; *t*_851_ = 1.2, *p* = 0.22, effect size = 0.11 for PPC).

Generally, similar results were present for the R1R2 task (Figures 6C,D). BS units exhibited mean ROC values during the first delay period that were significantly elevated above chance (one sample *t*-test, *t*_351_ = 6.56, *p* = 1.89E-10, effect size = 0.35 for dlPFC; *t*_503_ = 10.4, *p* = 6.5E-23, effect size = 0.46, for PPC). For the NS units, mean ROC value reached statistical significance only for the PPC, whereas dlPFC was marginally above our threshold of statistical significance (one sample *t*-test, *t*_65_ = 1.91, *p* = 0.06, effect size = 0.24 for dlPFC; *t*_91_ = 4.3, *p* = 4.5E-5, effect size = 0.45, for PPC). For both areas, no significant difference was detected between mean ROC values of NS and BS during the delay period when compared to each other (*t*_416_ = 1.02, *p* = 0.31, effect size = 0.14 for dlPFC; *t*_594_ = 0.40, *p* = 0.68, effect size = 0.04 for PPC).

**Figure 7:**
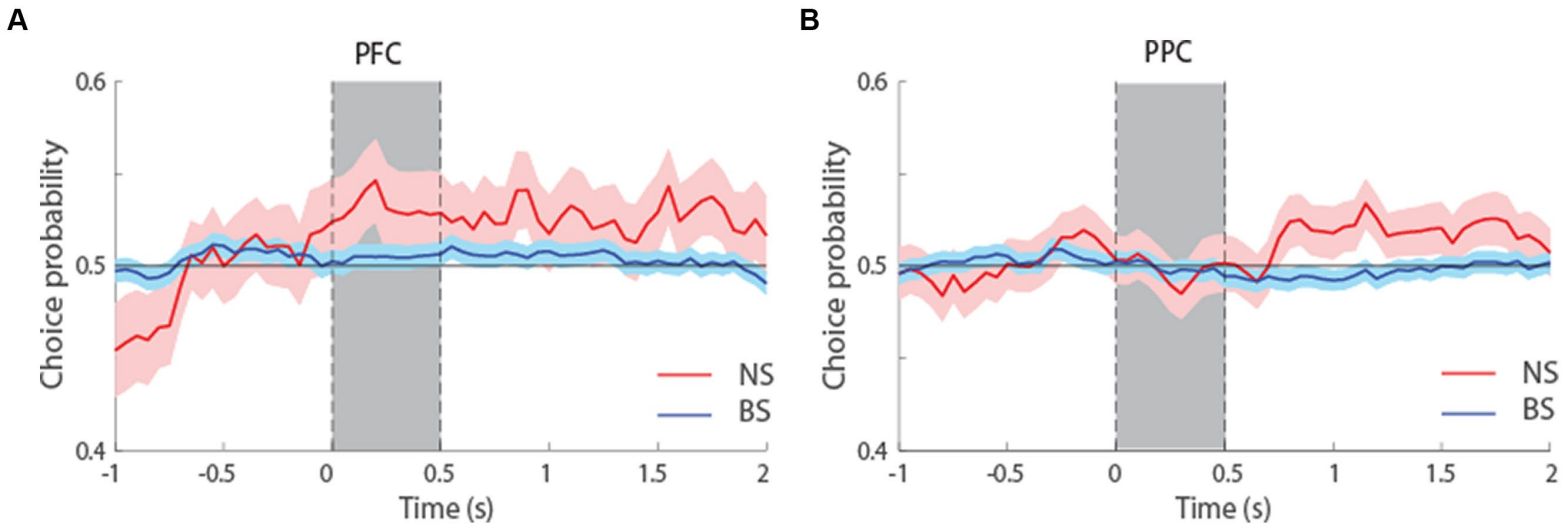
**(A)** Averaged area under the ROC curve from NS (red line, *n* = 65) and BS (blue line, *n* = 463) neurons recorded from the dlPFC, pooled from both the MSNG and R1R2 tasks. The solid line represents ROC value (choice probability) comparing the distribution of correct and error trials from the preferred cue condition. The shaded area represents SEM. **(B)** Averaged choice probability value from NS (red line, *n* = 117) and BS (blue line, *n* = 685) neurons recorded from the PPC, pooled from both the MSNG and R1R2 tasks.

We additionally performed an ROC analysis that compared the distributions of firing rates in correct and error trials, yielding a quantity sometimes referred to as choice probability (Britten et al., 1996). This analysis only included neurons with error trials at the condition that represented each neuron’s preferred location. To have sufficient power for comparisons and considering the overall similarity of ROC profiles for the two tasks (Figure 7), we pooled data from both tasks together and examined choice probability for the first 1.5 s of the delay period, which was common in both tasks. The results suggested higher mean Choice Probability values for NS than BS neurons in both tasks, and this difference reached statistical significance in the PPC (*t*-test, *t*_526_ = 1.9, p = 0.06, effect size = 0.23 for dlPFC; *t*_800_ = 2.02, *p* = 0.04, effect size = 0.19, for PPC). In other words, if the activity of neurons was lower than average in a trial during the delay interval, the subject was more likely to make an erroneous than correct choice and this relationship was stronger for NS than BS neurons, particularly in the PPC. Additionally, there was no significant difference in the Choice Probability values between NS neurons of dlPFC and PPC (*t*-test, *t*_180_ = 0.56, *p* = 0.58, effect size = 0.09), and between the BS neurons of dlPFC and PPC (*t*-test, *t*_1146_ = 1.21, *p* = 0.22, effect size = 0.07). The result suggests similar characteristics of both neuronal populations in these two areas, in terms of how their firing rate relates to behavioral outcome.

## Discussion

Persistent activity is considered the neural correlate of working memory (Constantinidis et al., 2018) but continues to be debated (Stokes, 2015; Lundqvist et al., 2016). The bump-attractor computational model suggests that persistent activity is sustained by virtue of recurrent connections between neurons with similar tuning, thus allowing discharges to reverberate in the circuit and be maintained stably even after the actual stimulus is no longer present (Compte et al., 2000; Wang, 2001; Murray et al., 2017a). Structured excitatory and inhibitory connections are both essential in the maintenance of working memory in this model (Constantinidis et al., 2002; Wang et al., 2004). Persistent activity has been shown to predict working memory performance, however evidence linking behavior with persistent activity based on delayed response tasks has been criticized as it may represent motor preparation rather than working memory per se (Lundqvist et al., 2018; Miller et al., 2018). Studies of working memory in non-visual modalities provide strong evidence against this interpretation (Romo and Salinas, 2003; Constantinidis, 2016). Alternative visuospatial working memory tasks have also been introduced, such as the MSNG task, which requires a categorical judgment about two stimuli and decouples the stimulus that needs to be maintained in memory from the eventual response. Our current results confirm this model prediction and demonstrate that narrow spiking, putative interneurons are active during several working memory tasks and generate persistent activity selective for the stimulus to be remembered even in tasks that decouple the memory for the stimulus location from response preparation.

Neuronal firing rate deviations during the delay interval of the task have been found to be predictive of subject recall (Li et al., 2021). Furthermore, correct and error trials differ in their level of delay period activity (Funahashi et al., 1989; Zhou et al., 2013). Although in theory, activity elevated above the baseline is not necessary for the encoding of stimulus information, neurons that generate persistent activity have been shown to be more informative about the remembered stimulus than neurons that respond to stimuli but do not generate persistent activity (Mozumder and Constantinidis, 2023; Thrower et al., 2023).

Our current results show that the activity of NS neurons that generate persistent discharges is also predictive of behavior, no less than BS neurons. Classification of cells into putative pyramidal neurons and interneurons is not perfectly precise and this determination cannot be made perfectly accurately for any single neuron, however neurons classified as NS are more likely to correspond to interneurons, allowing for meaningful comparisons between populations (Constantinidis and Goldman-Rakic, 2002).

### Roles of NS and BS neurons in cognitive functions

Classification of neurons into Narrow Spiking and Broad Spiking based on extracellularly recorded action potential waveform corresponds only imprecisely with inhibitory interneurons and pyramidal neurons, respectively. For example, some types of pyramidal motor neurons are known to exhibit short action potentials (Vigneswaran et al., 2011) and waveforms of excitatory and inhibitory interneurons in the mouse inferior colliculus have largely overlapping durations (Ono et al., 2017). In the monkey prefrontal cortex, comparisons of protein expression with action potential duration have shown that the majority of parvalbumin and somatostatin- expressing interneurons show narrow spike widths (Ghaderi et al., 2018; Torres-Gomez et al., 2020). Therefore, conclusions about differences between these types are meaningful but only at the population level.

Narrow Spiking and Broad Spiking neurons are coactivated in a range of cognitive functions, however their properties and roles are often divergent. Prefrontal Broad Spiking neurons generally exhibit smaller receptive fields, or sharper tuning for stimulus properties, than Narrow Spiking neurons (Constantinidis and Goldman-Rakic, 2002; Viswanathan and Nieder, 2017). BS and NS neurons are also differentially activated in attention tasks (Hussar and Pasternak, 2009; Ardid et al., 2015), with NS neurons demonstrating greater sensitivity to the stimulus dimension being attended to for the purposes of the task (Hussar and Pasternak, 2009). NS discharges are also synchronized to different frequency bands of the local field potential in the context of such tasks (Ardid et al., 2015). Dopamine, whose neuromodulating action has been implicated in the maintenance of working memory (Ott and Nieder, 2019), preferentially excites BS neurons and has been shown to suppress NS neuronal responses (Jacob et al., 2013, 2016). NS and BS neurons are also differentially affected by training to perform a new cognitive task, with NS firing rate elicited by stimulus presentations increasing to a greater extent (Qi et al., 2011; Qi and Constantinidis, 2013).

These results suggest that considerable specialization and division of labor in the context of different tasks exists between pyramidal neurons and interneurons (Wang et al., 2004). Our current results qualify these findings. We found that despite any specialization that may be present, NS neurons exhibit persistent activity that is informative about the stimulus location, and furthermore their activity is predictive of behavior consistent with predictions of the bump attractor model. Our results are in agreement with other studies as well, which have showed similar properties of NS and BS neurons during working memory (Torres-Gomez et al., 2020).

### Prefrontal and parietal roles in working memory

Numerous studies have implicated the prefrontal cortex as being critical for the maintenance of working memory (Constantinidis and Klingberg, 2016). However, evidence has accumulated of other brain areas playing a role in working memory, including the PPC, to the extent that is commonly referred to as part of the fronto-parietal network (Salazar et al., 2012; Murray et al., 2017b). Similar patterns of activation have been observed across dlPFC and PPC areas during working memory tasks (Qi et al., 2015). Studies have thus emphasized that cognitive performance relies heavily on a robust, distributed neural network across both areas (Chafee and Goldman-Rakic, 2000; Mejias and Wang, 2022).

In our current study, the activity of PPC neurons, at least in the first delay interval of both tasks, predicted the eventual behavioral choice of the subjects no worse than dlPFC neurons, as evidenced by the choice probability analysis. Prefrontal and parietal neurons do exhibit some specialization. PPC neurons are generally less able to resist the effect of distracting stimuli (Constantinidis and Steinmetz, 1996; Qi et al., 2010), though the specific patterns of responses in the two areas depend on the exact task a subject is performing (Jacob and Nieder, 2014). Persistent activity in prefrontal cortex also appears more robust and less variable from trial to trial (Qi and Constantinidis, 2015; Masse et al., 2017). Such differences in functional properties can be traced to differences in intrinsic properties of neurons and circuits in the two areas, including the relative frequency of different interneuron types in the prefrontal cortex (Zhou et al., 2012; Katsuki et al., 2014; Hart and Huk, 2020; Torres-Gomez et al., 2020). Despite these well-established differences, NS neurons in our sample generally behaved in a very similar fashion in the dlPFC and PPC. This suggests that fairly subtle activity changes are responsible for specialization between areas.

## Data availability statement

The original contributions presented in the study are included in the article/supplementary material, further inquiries can be directed to the corresponding author.

## Ethics statement

The animal study was approved by the Wake Forest University Institutional Animal Care and Use Committee. The study was conducted in accordance with the local legislation and institutional requirements.

## Author contributions

RM: Conceptualization, Data curation, Formal analysis, Investigation, Methodology, Project administration, Software, Validation, Visualization, Writing – original draft, Writing – review & editing. SC: Conceptualization, Data curation, Formal analysis, Investigation, Methodology, Software, Visualization, Writing – original draft, Writing – review & editing. SL: Data curation, Methodology, Writing – review & editing, Conceptualization, Investigation, Writing – original draft. CC: Conceptualization, Data curation, Formal analysis, Funding acquisition, Investigation, Methodology, Project administration, Resources, Supervision, Validation, Visualization, Writing – original draft, Writing – review & editing.
